# A Research Agenda for Helminth Diseases of Humans: Basic Research and Enabling Technologies to Support Control and Elimination of Helminthiases

**DOI:** 10.1371/journal.pntd.0001445

**Published:** 2012-04-24

**Authors:** Sara Lustigman, Peter Geldhof, Warwick N. Grant, Mike Y. Osei-Atweneboana, Banchob Sripa, María-Gloria Basáñez

**Affiliations:** 1 Laboratory of Molecular Parasitology, Lindsley F. Kimball Research Institute, New York Blood Center, New York, New York, United States of America; 2 Molecular Veterinary Parasitology, Department of Virology, Parasitology and Immunology, Faculty of Veterinary Medicine, Ghent University, Merelbeke, Belgium; 3 The Nematode Functional Genomics laboratory, La Trobe University, Victoria, Australia; 4 Council for Scientific and Industrial Research, Department of Environmental Biology and Health, Water Research Institute, Accra, Ghana; 5 Tropical Disease Research Laboratory, Division of Experimental Pathology, Department of Pathology, Faculty of Medicine, Khon Kaen University, Khon Kaen, Thailand; 6 Department of Infectious Disease Epidemiology, School of Public Health, Faculty of Medicine, Imperial College London, London, United Kingdom; McGill University, Canada

## Abstract

Successful and sustainable intervention against human helminthiases depends on optimal utilisation of available control measures and development of new tools and strategies, as well as an understanding of the evolutionary implications of prolonged intervention on parasite populations and those of their hosts and vectors. This will depend largely on updated knowledge of relevant and fundamental parasite biology. There is a need, therefore, to exploit and apply new knowledge and techniques in order to make significant and novel gains in combating helminthiases and supporting the sustainability of current and successful mass drug administration (MDA) programmes. Among the fields of basic research that are likely to yield improved control tools, the Disease Reference Group on Helminth Infections (DRG4) has identified four broad areas that stand out as central to the development of the next generation of helminth control measures: 1) parasite genetics, genomics, and functional genomics; 2) parasite immunology; 3) (vertebrate) host–parasite interactions and immunopathology; and 4) (invertebrate) host–parasite interactions and transmission biology. The DRG4 was established in 2009 by the Special Programme for Research and Training in Tropical Diseases (TDR). The Group was given the mandate to undertake a comprehensive review of recent advances in helminthiases research in order to identify notable gaps and highlight priority areas. This paper summarises recent advances and discusses challenges in the investigation of the fundamental biology of those helminth parasites under the DRG4 Group's remit according to the identified priorities, and presents a research and development agenda for basic parasite research and enabling technologies that will help support control and elimination efforts against human helminthiases.

## Introduction: Helminth Biology and the Prevention and Control of Helminth Infection

This century has seen a substantial global impetus towards raising public and scientific awareness of neglected tropical diseases (NTDs) in general and helminthiases in particular, and a great concerted effort has been made to elevate their political and funding profiles [1], [2]. As a result, the control of their morbidity and transmission has become highly important in the agenda of many public–private partnerships (PPPs) and national governments. The reasons for this, as well as the descriptions of the main ongoing initiatives against human helminthiases, are described in other reviews of this collection [3], [4]. Such initiatives have been partly fuelled by, or have themselves facilitated, much advancement in our understanding of the biology and epidemiology of the helminthiases they aim to control, and implementation has been followed by considerable success in many endemic areas. Sustaining this success and extending it to other more challenging situations brings a new set of questions, for which basic and operations research is urgently needed. In the context of this paper (and others in this collection), operations research is used to refer to the utilisation of relevant biological knowledge and appropriate and updated technologies by large-scale parasite control initiatives for the deployment of effective and optimal strategies aimed to reduce the parasite burden, transmission, and morbidity of poverty-related infectious diseases in general and helminthiases in particular.

Undoubtedly, successful and sustainable intervention against human helminthiases depends on optimal utilisation of available control measures and development of new tools and strategies, as well as on an understanding of the evolutionary implications of prolonged intervention on parasite populations and those of their hosts and vectors. This will depend largely on updated knowledge of relevant and fundamental parasite biology. On the one hand, it is reassuring that current interventions are mostly based on the application of past research, highlighting the important role that basic and operations research has indeed played in control programmes [3]. On the other, it is of concern that the research and development (R&D) agenda is not moving ahead at the pace required by the renewed impetus against helminth diseases. This is best illustrated by the reliance on single or very few drugs, most of which have been in use for many years (decades in some cases), none of which are highly efficacious in all settings and for all helminth species, and for which optimal dosages, combinations with other pharmaceuticals, and frequency of administration have not yet been established [5]. Additionally, more often than not, their mode of action is incompletely understood. For a recent review on unresolved issues in the pharmacology of anthelmintics we refer the readers to the authoritative paper of Geary et al. [6]. Likewise, assessment of infection at the individual and population levels relies on diagnostic tests that are in some cases older than the drugs, and whose diagnostic performance may not be the most appropriate, as parasite load and prevalence decline upon intervention [7]. Although more than one-third of the world's population is plagued by helminthiases [8], very little is understood in terms of host–parasite interactions and intra- and interspecific parasite interactions.

One of the main factors associated with the existence of research gaps in basic helminth biology is the understandable priority given to applied activities at the expense of basic research. “Understandable” because the imperative of controlling helminth infections, or relieving the morbidity associated with these infections, has led to the prioritization of deploying the interventions, and particularly delivering the drugs via mass drug administration (MDA); treating an increasing number of populations at risk, and ensuring high coverage. Since this has been effective in many cases, there has been a tendency to place support for more fundamental research on the back burner. However, as already discussed in this collection [5], reliance on MDA with a handful of drugs, and lack of knowledge of how parasite population and genetic structure will change under chemotherapeutic pressure, makes the control programmes potentially vulnerable to the development of drug resistance, particularly when few or no alternative drugs exist or are being developed (reviewed in [6], [9]). Furthermore, there is the potential for unintended consequences of MDA and other current or future interventions stemming from the poorly understood dynamics of host–parasite interactions, parasite–parasite interactions, and the changes that altering the parasite abundance (infection intensity and prevalence) of targeted species may have on those interactions. Not only will interventions have epidemiological effects, but they also will have evolutionary implications. In addition to the possible development of anthelmintic resistance, any measure that reduces the fitness of the parasite population in terms of its survival, reproduction, and/or transmissibility, will exert some selective pressure to which the parasites may respond adaptively [10].

Basic biology research should inform and underpin the prevention and control of helminth infection. The possible list of basic research issues is very long indeed and other authors before us have compiled extensive research agendas for specific infections [11], [12], but there are four broad areas that stand out as central to the development of the next generation of helminth control measures: 1) parasite genetics, genomics, and functional genomics; 2) parasite immunology; 3) (vertebrate) host–parasite interactions and pathogenesis; and 4) (invertebrate) host–parasite interactions and transmission biology. Box 1 lists the abbreviations used in this paper. We summarise recent advances and identify challenges in the investigation of the fundamental biology of helminth parasites of humans according to these four priority areas (Box 2), and present an R&D agenda for basic parasite research and enabling technologies that will help support control and elimination efforts, according to the deliberations of the Disease Reference Group on Helminth Infections (DRG4), established in 2009 by the Special Programme for Research and Training in Tropical Diseases (TDR) . The priority research areas identified are all grounded in a continuous effort to improve and update knowledge of helminth basic biology and to translate such knowledge into optimised intervention tools, and in so doing help to bridge the gap between the bench, clinical and population-based research studies, and operational programmes.

**Box 1. List of Abbreviations**

**BLAST,** basic local alignment search tool

**DRG4,** Disease Reference Group on Helminth Infections

**dsRNA,** double-stranded RNA

**ES,** excretory-secretory

**HUVE,** human umbilical vein endothelial cell proliferation

**IFN-γ,** gamma interferon

**IFNGR1,** IFN-γ receptor 1 gene

**IL,** interleukin

**LECs,** lymphatic endothelial cells

**LF,** lymphatic filariasis

**L3,** infective third-stage larvae

**MDA,** mass drug administration

**mf,** microfilariae

**NCC,** neurocysticercosis

**NTDs,** neglected tropical diseases

**OCP,** Onchocerciasis Control Programme in West Africa

**PPP,** public-private partnership

**R&D,** research and development

**RNAi,** RNA interference

**STHs,** soil-transmitted helminthiases

**TDR,** Special Programme for Research and Training in Tropical Diseases

**Th,** T helper

**WHO,** World Health Organization

**Box 2. Five Summary Points for Basic Research and Enabling Technologies for Helminthiases**

- Four areas have been identified in which basic research can contribute potentially to develop enabling technologies for successful parasite control. These are:
  1. Parasite genetics, genomics, and functional genomics
  2. Parasite immunology
  3. (Vertebrate) host–parasite interactions and pathogenesis
  4. (Invertebrate) host–parasite interactions and transmission biology
- Genomes of helminth parasites are becoming increasingly available and promise to revolutionise (also through related advances in transcriptomics and proteomics) the field of helminth biology and help unravel new targets for control. Without annotation and functional genomic tools, these data will not be truly useful to support the search for novel interventions. Knowledge of how parasite population genetic structure will change under chemotherapeutic pressure is essential to understand the evolutionary implications of intervention.
- Helminths have evolved to evade or subvert powerful, immune-mediated, host defense mechanisms. However, the processes that initiate and sustain immune regulation on the one hand, or lead to pathogenesis on the other, and the effects upon them of prolonged anthelmintic intervention remain incompletely understood.
- Knowledge of factors controlling host–parasite interactions can ultimately support identification of vulnerable pathways to be targeted by novel interventions and help avert unintended consequences of intervention (e.g., increased transmission, and/or morbidity).
- Vector/intermediate host–parasite interactions are usually under-appreciated, though they may hold the key to many of the epidemiological and evolutionary underpinnings of helminth infections with complex life cycles. Their improved investigation will help support the deployment of antivectorial control measures and understand the effects of these measures on parasite abundance and transmission dynamics.

## Current Advances in Basic Research on Helminth Biology and Future Challenges

### 1) Parasite Genetics, Genomics, and Functional Genomics

The onset of the genomics revolution has raised hopes for the development of applications in the field of human health. New tools addressing pathogens and their vectors have increased our understanding of evolutionary processes and the delicate interplay between parasites and hosts and with their environment. We can expect important technological advances, not only in new diagnostics, therapeutics, and vaccine development, but also in our understanding of disease mechanisms, host–parasite interactions, and transmission biology [13], [14]. In this regard, a comparison of helminthiases with malaria may be helpful in highlighting how these areas of research can transform a whole field. Malaria genomics over the past decade has reinvigorated drug and vaccine development, enabling the development of more comprehensive mathematical models to inform control and elimination efforts [15].

The recent research landscape for helminth parasites has been dominated by rapid progress in genome sequencing of several nematode and trematode parasites of significance to human disease. Today, the genome sequences of 22 species of helminths that either infect humans, or are closely related parasites, are completed or under way, including most or all of the significant soil-transmitted helminthiases (STHs), schistosomes, and filarial species [13]. A comprehensive genome analysis has been published for several of them, including the lymphatic filarial nematode *Brugia malayi* [16], and the blood flukes *Schistosoma mansoni* [17] and *S. japonicum* [18]. The recently published draft genome of the porcine parasite *Ascaris suum* also provides a comprehensive resource to study human ascariasis [19]. The cost of sequencing using second generation technologies is such that obtaining a genome sequence is no longer prohibitively expensive or seen as a major barrier or significant investment. Importantly, the genomes of *Loa loa*, *Wuchereria brancrofti*, and *Onchocerca volvulus* (http://www.broadinstitute.org/annotation/genome/filarial_worms/MultiHome.html) now have been sequenced in part as well. However, some of the available genomes are incomplete, poorly annotated, or not annotated at all, and there are almost no tools available with which gene function can be tested directly. Although the current genome drafts of *S. mansoni* and *S. japonicum* achieve 5- to 6-fold coverage of the entire genome, this includes large numbers of incontinuous contigs and supercontigs with gaps [20]. Without annotation and functional genomic tools, the sequence data are not truly useful, so careful thought should be placed to invest not only on sequence generation but also on annotation, capitalising on genome-wide approaches to understand the structure of parasite populations [21], [22]. However, it can also be argued that a good transcriptomic dataset is more useful for vaccine/drug screening, requiring substantially less investment in personnel (annotation expertise), a factor that slows down the genome sequence annotations. Examples of large transcriptomic studies from human helminths include those on *Necator americanus* [23], *S. japonicum* [24], *Clonorchis sinensis*, and *Opisthorchis viverrini* [25], [26], which provide very useful information for all the “omics” and discovery of vaccine antigens and drug targets.

The limited availability of functional genomic tools (with which gene function can be investigated) for helminths contrasts markedly with, for example, the situation with several groups of protozoan parasites (especially *Plasmodium* spp.), for which the development of functional genomic tools has accompanied genome sequencing (for a more detailed explanation of functional genomic tools in parasite research, see Box 3). This has resulted in much useful annotation of protozoan parasite genomes, which have yielded information that has been applied to practical ends, such as the creation of a comprehensive database containing a list of all potential drug targets for malaria (http://www.bioinformatics.org/mdt). Moreover, genome sequencing, accompanied by the annotation of protozoan parasite genomes and the subsequent development of functional genomic tools, has also enabled the generation of testable gene function hypotheses. The results of such experimental tests of function are now being applied to parasite genetic investigation, and drug and vaccine development. A similar, genome-driven expansion of schistosome research is gathering momentum as a result of developments that have followed the publication of annotated schistosome genomes and concurrent development of better tools with which those genome sequences can be utilised (http://www.genedb.org/Homepage/Smansoni; http://schistodb.net) [27]. However, despite the significant advances made in genomic, proteomic, and transcriptomic profiles of helminths, these “-omics” are still in the early developmental stages. Provided that effective functional genomic tools, similar to those already in use in malaria research, are developed also for helminths, available genomic data will have a major impact in the long term to support basic research that is needed if new treatments are to be developed and current ones made more effective and sustainable. Notwithstanding the obvious value of learning from successes with malaria parasites, *Toxoplasma gondii*, and other non-metazoan pathogens, the human helminths are much more complex organisms (e.g., they are diploid, have reproductive and other organs, nervous systems, etc.); there are many more species of them; they belong to two completely unrelated phyla (Platyhelminthes and Nematoda); no cell lines are available; the developmental cycles cannot be completed in vitro, and their developmental cycles are not only dissimilar to those of malaria and other apicomplexans, but they are also generally dissimilar to each other's, e.g., *A. lumbricoides* versus *W. bancrofti* versus *Echinococcus multilocularis*, etc.

**Box 3. Functional Genomic Tools and Helminth Research**

Functional genomic tools fall into two broad categories: 1) bioinformatic tools for sequence mining to generate hypotheses concerning likely biological function, and 2) experimental tools with which gene expression can be manipulated in the target organism (or, in the case of parasites, also the host) and the consequences of that manipulation for the biology of the parasite and its relationship with the host can be observed and measured.

The first bioinformatic tools that are applied are generally genome-wide homology searches, usually using variants of basic local alignment search tool (BLAST) to generate automatic annotations based on sequence homology. While perhaps useful as a tool with which to assess genome content, homology-generated gene annotations are at best a very rough guide and at worst downright misleading. Additionally, in parasites the limited utility of a homology-based approach is undermined further by the poor performance of gene-finding software in parasite genomic sequences. Nonetheless, several relatively advanced bioinformatic tools with which, for example, functional classes can be grouped or putative metabolic pathways predicted have been published recently along with examples of their application [27], [167]. These could be used to, for example, search for likely differences between the parasite and its host that may offer the opportunity for either vaccine or drug development, or to search for molecules that may mediate host pathology [13]. Allied to these bioinformatic tools is the dramatic increase in sequencing capacity such as deep “whole transcriptome” sequencing, which yields quantitative as well as qualitative data on parasite gene expression. These data will aid in gene finding and annotation as well as point to key regulatory events in the parasites' relationship with the host.

The most important functional genomic tool with which gene function can be investigated is RNA interference, whereby gene expression is knocked down by exposing the parasites to gene-specific dsRNA or siRNA. Unfortunately, the efficiency of RNAi in helminths varies between species [28]. Problems often arise with the efficiency, specificity and reproducibility of some methodologies, especially with nematode species. This clearly highlights the need for future research to optimise the delivery methods and culture systems of these parasites. More recently, transgenesis was established in some helminths whereby a transgene was introduced, both transient and heritable. At the moment, this is still used to overexpress certain transgenes, but in the long term it is hoped that this methodology can be used to silence genes by the introduction of antisense transgenes.

Regardless of such disparity, the development of tools with which parasite gene expression can be directly investigated has also been the subject of recent developments. RNA interference (RNAi, or gene silencing), whereby gene expression is knocked down (gene-specific double-stranded RNA [dsRNA] triggers degradation of homologous mRNA transcripts), has been attempted in several parasitic nematode and trematode species. The effectiveness of RNAi in helminths (particularly nematodes) seems to be somewhat variable, especially in nematodes, so it remains to be seen whether it will be a generally useful technique, or whether its application will be restricted to a handful of RNAi-susceptible species [28],[29]. For most parasitic nematodes (excepting *Haemonchus contortus*, a parasite of sheep), there are very few good (and reproducible) examples [30], [31]. Having said that, recent advances have been also made targeting the filarial nematode *B. malayi* as it develops in an intermediate host, the mosquito *Aedes aegypti*, thus supporting future parasitic nematode biology and the possibility of identifying and validating novel anthelmintic drug targets [32]. In contrast to the situation in nematodes, RNAi in schistosomes seems to be more robust and reproducible [33] and is currently being used by a number of groups to elucidate the function of some key proteins and pathways in these parasites, such as haemoglobin digestion [34], [35], tegument formation, and the biological role of tegumental proteins [36]–[39], and advances are being made for *S. haematobium* [40]. Additionally, a vector-based RNAi model for *S. mansoni* has recently been developed [41], [42]. These approaches have helped identify vaccine/drug candidates, some of which are in various stages of clinical development, providing examples of how bench research in a post-genomic era is revealing potential targets for novel interventions [43].

The alternative means by which gene function can be decreased is via loss of function mutation. The converse of knock-down of expression is manipulation of expression by gene knock-in. There are now several reports of either transient or heritable transgenesis of at least several species of parasitic nematodes and trematodes (three of which are parasites of humans) [13], [44], [45] and in at least one cestode, *E. multilocularis* [25]. The recent advances in transgenesis offer some hope of reverse genetic analysis via gene knockout. Other techniques that have been developed for helminths are whole-mount in situ hybridisation and microarray analyses [46]–[51], but their use is limited for functional analysis of helminth-encoded genes. We refer the readers to the recent review on transgenesis and gene delivery routes in parasitic nematodes by Lok [52].

Another field that will benefit from improved genomics and bioinformatics and novel enabling technologies is parasite population biology studies. In filarial species there have been difficulties in developing microsatellite markers, but in other species, such as in schistosomes, the generation and use of microsatellites has helped to understand transmission structuring in parasite populations according to environment and host species [53]–[55], as well as changes in genetic diversity under treatment [56], [57]. Population genetic studies of *Ascaris* have helped to understand transmission patterns within and between *A. lumbricoides* and *A. suum* [58]. Without a robust understanding of parasite population structure, it will be difficult to assess the short- and long-term evolutionary implications of anthelmintic interventions in general and chemotherapeutic pressure in particular. The few population genetic studies of helminths conducted to date suggest, not surprisingly, that the nature of the parasite's life cycle (i.e., direct or indirect transmission; transmission via biting arthropods or snails; single or multiple definitive hosts, etc.) has a very significant impact on parasite population genetics [59]. Furthermore, the population genetics of a given species may be different under different circumstances (e.g., schistosome populations in different epidemiological settings show different genetic structure [54], [60]). Mathematical modeling suggests that differences in population structure will affect transmission and, importantly, the selection and spread of drug resistance alleles (we refer the readers to Basáñez et al. [61] in this collection for a discussion of these aspects).

The major challenge here is that despite the acknowledged importance of parasite population biology and population genetics for understanding parasite transmission, relevant data are sparse for most helminth species. This is especially problematic when attempting to monitor the impact of control measures such as MDA on parasite populations, their structure and reproductive biology, the incidence of new infections and how this may be affected by the relaxation of any regulatory processes that may operate (including acquired immunity), and the potential for the selection and spread of resistant genotypes [59]. Once again, the experience from malaria, where the phenomena of drug resistance selection and its spread are well recognised, suggests that the development of tools with which to promptly monitor drug resistant genotypes, and deploy opportunely appropriate strategies in response to such selection, require detailed knowledge of parasite population structure and genetics. Acquisition of this knowledge for helminth parasites must be given high priority. Therefore, it is important that genome sequencing resources be applied also to the investigation of parasite population genetic structure, and the testing of predicted patterns of population sub-division and gene flow.

Research funding for translational basic research will follow the development of tools with which fundamental questions cannot only be posed, but also answered. What is required is “seed” funding to develop the genomic resources. The genomic data becoming available for schistosomes could have a major impact in the medium and long terms [22] provided that similar, effective functional genomic tools are developed for other helminths (especially for nematodes).

### 2) Parasite Immunology

Parasites and hosts interact primarily via the host immune system. Work over the past several years in parasite immunology, mainly in rodent models, has focused to a large extent on the identification of mechanisms of protective immunity that could shed light on helminth vaccine development, but the emerging theme in basic research is the realisation that the host–parasite immunological relationship is highly interactive, and that helminths are masterful immunoregulators [62], [63]. Immune regulation by parasites includes suppression, diversion, and alteration of the host immune response. Numerous studies have indicated that helminth-secreted proteins, glycoproteins, and lipid-based molecules can interfere with various arms of the host immune response, ultimately leading to the generation of an environment favourable to the parasites' survival [64]–[66]. Some of the processes affected include the development of allergic responses and interference with host cytokine regulation and signal transduction networks [67]–[69]. These findings highlight the complexity of helminth immunobiology with respect to the host–parasite interaction, which is further complicated by polyparasitism and potential inter-specific interactions, either by other helminths of the same or different groups, protozoan parasites, bacterial infections, or viral infections. Helminths have evolved to co-adapt with their hosts and to evade/subvert powerful host defense mechanisms, and it is these intricate interactions and evolutionary trade-offs that have made them such successful pathogens.

A characteristic feature of helminth infection is a T helper 2 (Th2)-dominated immune response, but stimulation of immunoregulatory cell populations, such as regulatory T cells and alternatively activated macrophages, is equally common. Typically, Th1/Th17 immunity is blocked and productive effector responses are muted, allowing survival of the parasite in a “modified Th2” environment. Successful immunoregulation also limits collateral damage to the host. The remarkable range of helminth life histories, transmission strategies, and physiological niches is reflected in the variety of immunomodulatory activities targeting key receptors or pathways in the mammalian immune system observed across the three taxa of nematodes, cestodes, and trematodes that comprise the helminth grouping [63]. However, the mechanisms that initiate and sustain this immune regulation remain incompletely understood. These immunoregulatory mechanisms are important not only in the context of explaining the characteristic chronic infections, the absence of protective immunity after first infection, and the difficulties faced in the attempts of developing anti-helminthic vaccines [70], but also for the evaluation of MDA and other treatment programmes. Chemotherapy-based programmes can alter the dynamics of transmission and the burdens of infection in treated communities and are therefore likely to perturb these immunoregulatory relationships, and thus have the potential to reverse these immunoregulatory effects. This change could also have unintended consequences for global elimination efforts, such as increased susceptibility to infection or to patency; increased disease burden in children (who would not have developed tolerising immune responses elicited by exposure to parasite antigens in utero, in contrast to their counterparts prior to control [71]; or increased morbidity due to the targeted helminth and/or other concurrent infections. Also, the strength and duration of immune responses are unknown, making it difficult to implement immunity-explicit mathematical models that could help predict the impact of anthelmintic treatment on reinfection and immunity parameters [61], [72].

The unintended immune consequences of treatment or other interventions destined to reduce infection load and/or incidence are not limited only to the targeted helminth infection. There is increasing evidence of the importance of co-infections, in which parasites and pathogens could interact in a synergistic or antagonistic fashion. Suppressing or removing one parasite species could give selective advantages to others by decreasing immune-mediated competition or inhibitory effects. For example, it has been proposed that individuals infected with parasitic helminths have increased susceptibility to malaria infection [73], [74], and that helminth infections may also alter susceptibility to clinical malaria [75], [76]. There is now increasing interest in investigating the consequences of such co-infection [77] and assessing whether mass deworming affects the incidence of clinical malaria or other infections, and such studies should be encouraged.

Concurrent helminth infections have been also shown to alter optimal vaccine-induced responses in the human host; however, the consequences of this condition have not been adequately studied, especially in the context of an infection following vaccination. Demands for new and effective vaccines to control chronic diseases like tuberculosis, HIV, and malaria as well as deploying vaccines for the so-called childhood diseases to all in Africa require a systematic evaluation of confounding factors that may limit vaccine efficacy, such as the presence of co-infections with helminths in the populations of humans targeted for vaccination. The bias towards a Th2 cytokine milieu induced by helminth infection, especially the notable depression of gamma interferon (IFN-γ), which is pivotal in cellular immune responses, has been compared to an “anti-adjuvant” effect [78]. It has been shown that the presence of helminths may alter host responses to bystander antigens like the tetanus toxin vaccines [79]–[82], probably due to polarisation of the immune response to a Th2-like response or the production of immunomodulating cytokines like interleukin (IL)-10 that dampen both Th1 and Th2 responses. The reduced response to the oral cholera vaccine observed in individuals with *A. lumbricoides* could be, however, restored by albendazole treatment [81]. The potential impact of helminth infections on novel tuberculosis and malaria vaccines trials will have to be considered [83], [84].

Most importantly, it must be remembered that most of the anthelmintics currently being used are not totally curative, and numerous rounds of MDA may be necessary to reduce the levels of infection below those necessary to sustain transmission [85]. Thus, it can be anticipated that such major alterations in the levels of infection in endemic communities might have a dramatic impact on the degree of their immunity to the targeted parasites and other co-infections, resulting in either a higher degree of protection against re-infection (thereby promoting success of the MDA), or conversely, resulting in less protection (and becoming a potential impediment to elimination). For example, studies in humans and cattle have shown that *Onchocerca*-infected hosts, in which infections were cleared by chemotherapy, acquired new infections of equal or higher intensity than those exhibited before the therapeutic intervention [86]–[88]. Therefore, for example, in areas where MDA with ivermectin does not result in transmission interruption, those who are re-infected might develop a higher burden of infection. Similar data exist for schistosomiasis and STHs [89]–[93]. A better understanding of the host–parasite immune relationships at play at the molecular level and at different life cycle stages within the host is thus important not only to make more precise predictions about the eventual success of the specific elimination efforts, but also to alert the MDA programmes of potential problems that might arise from altered immunity in treated communities.

### 3) (Vertebrate) Host–Parasite Interactions and Pathogenesis

Host responses to helminth parasites are important factors in disease manifestation. Typically, pathological characteristics may manifest initially as acute reactions that may be followed by chronic inflammation that results in significant immunopathology: much of the disease is due to the host's response to the presence of the parasite rather than the direct action of the parasite. Primary infection in naïve hosts often results in acute disease manifestation. For some parasites, as the infection moves from the initial acute phase to a chronic phase, inflammatory responses may resolve, leaving many patients asymptomatic, but in a proportion of patients (which varies in different host–parasite relationships) the acute initial phase is followed by chronic inflammation. These chronic inflammatory responses often do little or no damage to the parasites, and in the case of penetration of the eggs of *S. mansoni* through the intestinal wall, are actually exploited by the parasite to its advantage. Modulation of host immune responses by the parasites is a likely explanation for many of these phenomena, but the details of the transition from acute to asymptomatic versus chronic inflammation are generally unknown. The following sections summarise current knowledge on the pathogenesis of the infections under the remit of the DRG4 [3].

#### Onchocerciasis and lymphatic filariasis (LF)

In onchocerciasis, the interaction between *O. volvulus* and the host's defence system is vital to the individual's tolerance of the infection. Children born to mothers with *O. volvulus* had not only a substantially higher risk of becoming infected, but also acquired infection earlier in life and developed higher infection levels [94]. Different immune responses to *O. volvulus* cause considerable variation in the clinical manifestations of human onchocerciasis, from generalised to hyper-reactive onchocerciasis [95]. Onchocercal lesions result from inflammatory reactions involving immunological mechanisms. The role of the immune system in the pathology is demonstrated by the accelerated worm destruction (microfilarial stages) during microfilaricidal chemotherapy. Microfilarial destruction can be mediated by antibodies to the surface-associated antigens of the worm and enhanced by complement.

*O. volvulus* and the lymphatic filariae harbour intracellular *Wolbachia* bacteria, now recognised as obligatory endosymbionts essential for reproduction and survival of the worms, and therefore emerging as novel targets for chemotherapy [96]. The presence of *Wolbachia* may also be involved in immune evasion by the worms [97], but on the other hand, it has also been implicated in the immunopathogenesis of filarial infections [97]–[101]. Inflammatory responses following treatment of filarial infections with diethylcarbamazine or ivermectin have been suggested to result in part from the release of high numbers of endobacteria from degenerating blood or tissue microfilariae (mf) [102], [103]. *Wolbachia* and their products are reported to elicit pronounced innate immune responses in vitro consistent with those observed previously in treated filariasis patients [104]–[108]. Interestingly, differences in *Wolbachia* abundance between the savannah and forest forms of *O. volvulus* may help explain differences in ocular pathogenicity [109]. Now that both genomes and excretory-secretory (ES) proteomes of *B. malayi* and its *Wolbachia* are known [16], [110], [111], future studies will help to further understanding of *Wolbachia*'s role in pathogenesis prior to and after the introduction of MDA.

#### Soil-transmitted helminthiases (STHs)

Although the pathology due to these intestinal nematode infections is relatively well known (see the excellent seminar in [112]), the pathogenesis and pathogenic mechanisms of most STH infections have remained poorly elucidated. The number of specific virulence factors identified for each of the major parasite species is very scanty. Moreover, for those specific parasite genes and gene products thought to be important in infection and/or pathogenesis, it has been difficult to demonstrate a definitive role due to the inability to reliably silence gene expression in vitro or in vivo. Some advances have been made in understanding the role of human host genetics in the predisposition to STH infection [113], [114], and possibly these factors could also play a role in disease manifestations. This is therefore an area that requires further research efforts.

#### Schistosomiasis and other trematode infections

Exacerbation of host pathology occurs in a certain number of individuals with schistosomiasis, and this may be explained by host genetics/immunogenetics. In chronic schistosomiasis, severe hepatosplenic pathology occurs in less than 10% of the infected population. The pathology is characterised by excessive deposition of collagen and other extracellular matrix components around schistosome egg granulomas in the liver, causing periportal fibrosis and progressive occlusion of the portal veins [115]. In murine schistosomiasis, the pathology is induced by a CD4+ Th2-driven granulomatous response directed against schistosome eggs lodged in the host liver. The Th2 cytokines IL-4 and IL-13 drive this response, whereas IL-10, IL-13Rα2, IFN-γ, and a subset of regulatory T cells act to limit schistosome-induced pathology. A variety of cell types including hepatic stellate cells, alternatively activated macrophages, and regulatory T cells have also been implicated in the pathogenesis of schistosomiasis. Current knowledge suggests the immunopathogenic mechanisms underlying both urinary and intestinal schistosomiasis are likely to be similar [116]. Interestingly, a recent study has reported lower liver morbidity and higher bladder morbidity in mixed *S. mansoni*–*S. haematobium* infections compared to single *S. mansoni* infections, possibly explained by the localisation of the hybridising adults (*S. haematobium* males mating with *S. mansoni* females and the subsequent [infertile] eggs produced from such couplings passing to the urinary oviposition site, thereby reducing the amount of classical *S. mansoni*–induced morbidity whilst increasing the classic *S. haematobium*–associated bladder morbidity) [117].

Host genetic background also plays a pivotal role in determining the susceptibility to and outcome of schistosome infections [118]–[120]. For example, segregation analysis of a Brazilian population has revealed that susceptibility to infection is controlled by the *SM1* (*S. mansoni* 1) gene locus that has been linked to the 5q31–q33 chromosome region comprising the genes for IL-4, IL-5, and IL-13 [121], [122]. Another study involving a Sudanese population indicated that the segregation of a co-dominant gene (*SM2*) could account for the familial distribution of severe *S. mansoni* schistosomiasis in this population. Linkage analysis indicated that this gene occurred within the 6q22–q23 region with polymorphisms close to and in the IFN-γ receptor 1 gene (*IFNGR1*) [118]. A better understanding of factors that influence infection, pathology, and protection is needed.

Other liver flukes (*Fasciola hepatica*, *Opisthorchis* spp., and *Clonorchis sinensis*) use their suckers (oral and ventral) in hooking on biliary epithelium, causing ulceration, for nutrition and migration [123]–[125]. *Fasciola* proteases, some of which are developmentally regulated, can degrade host tissue and blood, and form abscesses during different migration phases, including intestinal wall or liver penetration [123], [126].

In other trematodiases, severe fibrosis of affected tissue/organs is also a hallmark of chronic infection in certain individuals. Advanced periductal fibrosis around the intra-hepatic bile ducts in people with *Op viverrini* is associated with elevated parasite-specific IL-6 production among 11 Th1/Th2 cytokines, in comparison to those with no or minimal fibrosis [127]. Moreover, the fibrosis occurs in a small subset of infected populations. Detailed studies on molecular pathogenesis mechanisms of the liver fluke trematodes need to be conducted (see for instance Smout et al. [128]).

#### Cestode infections

Cysticercosis, caused by *Taenia solium* larvae, is a major public health problem, especially in the developing world, and neurocysticercosis (NCC) is considered to be the most common parasitic infestation of the central nervous system [129], [130]. Approximately 25% to 50% of active epilepsy cases in the developing world, including India and Latin America, are due to NCC [131]. NCC induces neurological syndromes that vary from an asymptomatic infection to sudden death. Neuroimaging is the mainstay of diagnosis. The genome project of *T. solium* has been started [132] and knowledge of the genetic structure of *T. solium* is being applied to studies on the epidemiology, transmission, and pathogenicity of this disease [133]. Studies on innate and acquired immune responses in human *T. solium* NCC, which can persist for decades, have highlighted conditions that appear to be favourable for the survival or destruction of the parasite and for the benefit or injury to its host [134]. In addition, animal models for the immunology of cysticercosis in *T. crassiceps* infecting mice and *T. solium* infecting pigs add more information on immune regulation of cysticercosis. The parasite manipulates the host immune system to support its survival by keeping a low inflammatory profile caused by the production of some cysticerci-released products that have immunomodulatory activities [135]–[137]. Moreover, the mouse model has been used to design vaccine strategies, some of them with promising results [135]. Further research is needed to elucidate the role of the host's immune response in 1) developing an acute inflammatory response around the parasite, which is strongly associated with symptoms, and seems to mark the onset of the process of parasite death [138]; 2) developing peri-lesion oedema in old, calcified lesions (this is also strongly correlated with new symptomatic episodes) [139], [140]; and 3) controlling and eliminating infection, most likely in mildly exposed individuals [141]. Importantly, the mechanisms used by the parasite to modulate the host's immune system at the central nervous system level, and which allow its survival for years, also needs to be studied [136], [137].

#### Helminths as Group 1 carcinogens

In contrast to the view that helminth infections are generally associated with morbidity rather than mortality, the most severe pathology associated with some helminth infections is cancer. Chronic infections with *Op. viverrini* and *C. sinensis*, the Asian liver flukes, have long been associated with cholangiocarcinoma or bile duct cancer [125], [127], and experimental studies on the proliferative effects caused by E-S products of these species provide clues as to the mechanisms involved [142]–[144]. Analysis of transcriptomic datasets of *C. sinensis* and *O. viverrini* for proteins common to carcinogenesis identified a large number of proteins that are homologues of genes involved in human cancer development [25], [145]. It is anticipated that these transcriptomes will contribute significantly to the identification of novel intervention tools.

Helminth-associated cancer is, however, not restricted to Asian liver fluke infections. The eggs of *S. haematobium* provoke granulomatous inflammation, ulceration, and pseudopolyposis of the bladder and ureteral walls. Chronic lesions can then evolve into fibrosis, and carcinoma of the bladder (squamous cell carcinoma) [146]. All three of these helminth parasites have been designated as Group 1 carcinogens—metazoan parasites that are carcinogenic to humans—by the International Agency for Research on Cancer of the World Health Organization. Therefore, not only do these trematodes cause pathogenic helminth infections, but they also are carcinogenic in humans in a similar fashion to several other more well-known biological carcinogens, in particular hepatitis viruses, human papilloma virus, and *Helicobacter pylori*. Similarly, live filarial parasites or filarial antigens induce significant human lymphatic endothelial cell (LEC) proliferation. Moreover, serum from patently infected (mf-positive) patients and those with longstanding chronic lymphatic obstruction induced significantly increased LEC proliferation compared to sera from uninfected individuals [147]. Live, intact *S. mansoni* eggs secrete a soluble factor that stimulates human umbilical vein endothelial cell proliferation (HUVE) in vitro in a manner similar to crude soluble egg antigen [148]. So overall, several helminth proteins possess mitogenic effects on a variety of cells and may directly induce cell proliferation. However, the number of studies performed to unravel the mechanisms underlying the pathology seen in helminthiases such as lymphangiogenesis (LF), neovascularisation (schistosomiasis), or biliary proliferation and carcinogenesis (opisthorchiasis and clonorchiasis) are limited [128], [144], [149], [150].

### 4) (Invertebrate) Host–Parasite Interactions and Transmission Biology

Recent advances in transmission biology have been partly reflected in the development of mathematical models for parasite population and transmission dynamics (see Basáñez et al. in this collection [61]). Of all the helminthiases considered under the remit of DRG4 (see [3]), filarial nematode and trematode infections are the ones with complex life cycles involving a vector or a snail host, respectively. (Other complex cycles, including nematode life cycles that require molluscan hosts, are those of *Angiostrongylus cantonensis*, for instance, and other complex developmental cycles occur in other taxa such as *T. solium*, *Gnathostoma spinigerum*, and *Capillaria philippinensis*. Of the latter, only *T. solium* is under our remit, requiring a mammalian intermediate host.) This interface is not of trivial importance given the close biological association that exists between the parasites and their invertebrate hosts. Yet, vector/intermediate host–parasite interactions are usually under-appreciated, though they may hold the key to many of the epidemiological and evolutionary underpinnings of these infections.

#### Vector–filaria interactions

For the filariases, the issues involved are ingestion of the skin- or blood-dwelling mf, their survival and development into infective third-stage larvae (L3) (there is no multiplication of the parasite within the vector), and, in common with all vector-borne diseases, survival of the vector until completion of the extrinsic incubation period and beyond. These processes have been investigated from genetic, immunological, physiological, and ecological perspectives in an effort to understand the basis for susceptibility/refractoriness to the parasites by the corresponding arthropod taxa (e.g., mosquitoes in LF, blackflies in onchocerciasis), and the close association between vector biting behaviour and the availability of mf to be ingested from blood or skin. From a population biology point of view, some of these underlying processes translate into relationships between consecutive parasite life stages that are of interest for epidemiological models. Some of these relationships (e.g., the number of *O. volvulus* or *W. bancrofti* L3 larvae per fly or per mosquito as a function of the microfilarial load on which the insects were fed) may be nonlinear, indicating the operation of density dependence [151], [152]. It is apparent that density-dependent processes regulate parasite population abundance and their effect is relaxed as a result of anthelmintic treatment, leading to enhanced per parasite probabilities of transmission. Therefore, an understanding of vector–parasite interactions becomes even more crucial as control programmes progress from morbidity reduction to elimination goals. Also, in some settings, there may be various vector–parasite combinations whose features may be impacted differently by interventions (e.g., differential effects of antivectorial measures depending on whether vector species feed and rest indoors or outdoors, or have a propensity to feed on humans or non-human blood hosts). (See Griffin et al. [153] for a theoretical exploration of malaria transmission in Africa, but similar issues will arise, and will need research in LF, particularly where both infections are transmitted by the same *Anopheles* vectors.)

Very few of the vector–filaria combinations have been characterised in detail, including studies of geographical distribution, ecological requirements of insects' aquatic and adult stages, vector competence, vectorial capacity, and local adaptation, among others. Without these studies, detailed mapping of the distribution of vectors and parasites will remain elusive. Vector competence encompasses the processes by which the vectors locate, ingest, and allow the parasites to complete their extrinsic incubation period. For vectors to transmit, they not only must survive such a period but also beyond it (the so-called infective life-expectancy or longevity factor in vector-borne diseases) [154]. In general, these processes remain poorly characterised and quantified in those vector–filaria combinations that are responsible for transmission in endemic areas. Some effort has been placed in doing so for *Simulium*–*Onchocerca* complexes given the impetus of the former Onchocerciasis Control Programme in West Africa (OCP) and the need to quantify such relationships for their use in mathematical models [151]. Likewise, and in order to explore the likelihood of elimination in LF settings, statistical descriptions of mosquito–*Wuchereria* interactions have received attention [155], [156]. Less is known about natural mosquito–*Brugia* and tabanid–*Loa* interactions, knowledge of which relies on old descriptive studies that, although still relevant, would need to be updated and expanded. Vector competence studies should be complemented by vectorial capacity investigations. Vectorial capacity (a close relative of the basic reproduction ratio in vector-borne diseases) also includes factors such as vector to host ratio, vector biting rate on humans, the propensity of vectors to feed on human or non-human blood hosts, vector mortality, and any seasonal and/or spatial dependencies that may occur in these factors. Knowledge of these, as well as of any density dependence that may operate (e.g., on the density of vectors and/or hosts, on the density of parasites), will inform design and implementation of vector control and any transmission-blocking intervention that may be developed. Yet, knowledge is still very scarce as to if and how helminth parasites manipulate these crucial aspects of the interaction. There is increasing evidence that this is the case in malaria, but experimental and observational studies in filariasis have lagged behind. It will also be of great interest to conduct research on how the *Wolbachia* symbionts of arthropod vectors and the *Wolbachia* symbionts of the filarial nematodes influence the competence of the vectors and the transmission biology of the parasites.

#### Snail–trematode interactions

Schistosome transmission occurs via free-swimming larval stages, cercariae, infective to mammalian definitive hosts, and miracidia, infective to the molluscan intermediate hosts. These non-feeding larvae obtain their energy through limited glycogen reserves, so there are strong selective pressures to locate and penetrate a suitable host rapidly post-emergence. Some of the snail–parasite issues in the schistosome life cycle are similar to those of filariases, highlighting the importance of parasite survival and development into the infective stage, and the survival of the intermediate host. In addition, schistosome larvae must themselves locate their subsequent hosts and asexually reproduce within the snail. Schistosome miracidia have evolved effective snail-seeking behaviours [157], [158], e.g., *S. mansoni* miracidia show geonegative and photopositive responses whereas *S. haematobium* show geopositive and photonegative responses, directing them, respectively, towards their contrasting *Biomphalaria glabrata* and *Bulinus globosus* snail host habitats. Young/recently hatched miracidia of ∼1–3 hours exhibit dispersal strategies rather than host attraction [159], potentially limiting density-dependent constraints occurring in the snail hosts. Density trade-offs appear to occur throughout the parasites' life cycle [160]. Miracidia have also been demonstrated to show sympatric specificity for host location [161] and penetration [162].

Once successful penetration of a suitable snail host has occurred, schistosomes undergo migration, asexual reproduction, and emergence of cercariae whilst evading the snail's immune response. Trade-offs often occur between daily cercarial production and host longevity, life-history traits, and virulence, with lower daily shedding associated with higher host survival and longer infectivity [160]. Intra- and inter-specific interactions also affect life-history responses, with *S. mansoni* mixed-strain infections inducing greater snail mortality in comparison to single-strain infections [163].

Cercarial emergence can vary in its chronobiological rhythm to maximise the chances of encountering a suitable definitive host. These interactions are also affected by inter-specific and intra-specific variation [164]. Lu et al. [165] have shown that in *S. japonicum*, which can infect up to 40 definitive host species, cercarial emergence from rodent infections peak at dusk and dawn, when their hosts are most active, whilst cercarial emergence for bovine strains peaks at noon.

Schistosome snail hosts are hermaphroditic, so even with intense mollusciciding or drought, they can still repopulate from extremely low numbers. However, the host-specificity shown by the Egyptian strains may have contributed to explaining why mollusciciding alongside mass praziquantel treatment was successful, with no long-term resistance emerging within Egypt [166]. Thus, an understanding of snail–schistosome interactions may be crucial for identifying optimal control mechanisms.

Regarding the snail–trematode interactions, most of the efforts have been made for the schistosomes as described above, but the literature on the ecological, evolutionary, and epidemiological relationships between the other trematodiases referred to in this report and their snail hosts is sparse. It would seem that the reference to “food-borne trematodiases” (because of the nature of the subsequent intermediate hosts for these parasites) has somewhat decreased the importance of their first, snail hosts. Yet, it is within the snail hosts that many important processes allowing the asexual multiplication of the parasite take place. Unlike schistosomes, the eggs of *Clonorchis* and *Opisthorchis* are eaten by the snail hosts (rather than the miracidia locating and invading the snails), but the resulting cercariae also need to find and locate their second intermediate, freshwater fish host. These are not just passive transport hosts of the parasites as it is in the fish that the metacercariae (the stages infective by digestion of raw, undercooked fish) develop. Therefore, the study of the host–trematode interactions for these infections must include those taking place in the snails and the vertebrate intermediate hosts. Difficulties in maintaining the entire life cycle of these parasites in the lab would certainly impose constraints on experimental studies such as those conducted for schistosomes.

## Concluding Remarks and Recommendations

From the discussion above, we propose an R&D agenda for basic helminth biology that is summarised in Box 4 and expanded in Text S1. This R&D agenda will support the development of new intervention tools and control measures for helminth infections. It is our contention that the success of present and future intervention programmes will require basic research that will help to bridge the gap between the bench, the clinical and population-based research studies, and the operational programmes. The recent research landscape for helminth parasites has been dominated by rapid progress in genome sequencing of several nematode and trematode parasites of significance to human disease. Future genome-wide analyses will support efforts to elucidate the basic biology of helminths, including immune-mediated and other host–parasite interactions that are relevant to helminth diseases of humans. They will help to develop novel intervention strategies such as drugs and therapeutic or prophylactic vaccines, as well as to identify parasite biomarkers and devise improved diagnostics. As many of the helminth infections are transmitted by arthropod vectors or involve intermediate hosts, a greater understanding of the interaction between vector/intermediate hosts and parasites is also important. Such research may be useful to identify potential targets for parasite growth and survival within the vector and transmission to the human host.

**Box 4. Five Key Recommendations for a Research and Development Agenda for Basic Helminth Biology**

1. Complete parasite genomes, annotate genomes and transcriptomes, and develop tools for parasite functional genomics in key species. Also use these tools to investigate parasite population and genetic structure, as this will be crucial for understanding how this structure will change under intervention pressure.
2. Identify the mechanisms involved in host immune responses to helminth infections, including both protection and pathology, as well as the mechanisms by which the parasites regulate such process to maximise their survival and reproduction. Translate knowledge of these mechanisms and their evolutionary trade-offs into rational strategies for vaccine development and deployment.
3. Investigate how helminth parasites modulate host–parasite interactions at the population and within-host levels, including the impact on the host immune response of concurrent infection with other helminth and non-helminth pathogens, the impact of parasite control interventions on such host–parasite interactions, and how concurrent infections affect clinical outcomes and the host's ability to seroconvert upon vaccination.
4. Define the determinants and mechanisms of helminth-associated pathology, including carcinogenesis and other processes involve in the association between helminth infection and excess human mortality.
5. Investigate the mechanisms underlying intermediate host/vector–parasite interactions and their ecological, epidemiological, and evolutionary implications. An understanding of vector–parasite interactions will become even more crucial as control programmes progress from morbidity reduction to elimination goals and will also be essential for the design of new approaches to vector/intermediate host control.

## Supporting Information

Text S1 Recommendations to policy and decision makers: identification of priorities for basic helminth research and the development of enabling technologies to support helminthiasis control and elimination.
(DOCX)
