# Supplementary material for: A Research Agenda for Helminth Diseases of Humans: Basic Research and Enabling Technologies to Support Control and Elimination of Helminthiases
Source: PLoS Negl Trop Dis. 2012 Apr 24;6(4):e1445. doi: 10.1371/journal.pntd.0001445 (PMC3335859; doi:10.1371/journal.pntd.0001445)
Supplement: Text S1 — Recommendations to policy and decision makers: identification of priorities for basic helminth research and the development of enabling technologies to support helminthiasis control and elimination. (DOCX) [file pntd.0001445.s001.docx]

**Supplementary Text S1. Recommendations to policy and decision makers: identification of priorities for basic helminth research and the development of enabling technologies to support helminthiasis control and elimination**

To realize the full potential of basic research on helminth biology for the prevention and control of helminth infections of humans the following will be necessary (see Box 2 in the main text):

**1. Parasite Genetics, Genomics and Functional Genomics**

- Complete reference genomes for the major helminth species affecting humans and develop tools to annotate helminth genomes
- Develop functional genomic tools with which to test the predictions that arise from those annotations and to study genes in nematodes, trematodes, and cestodes
- Study genomics/transcriptomics/proteomics of the parasitic worms, including genetic manipulation of parasites to determine the importance of new target interventions and to identify molecules that are essential for critical stages of parasite development
- Apply next generation sequencing technologies to analyze gene expression and regulation throughout helminth lifecycles and develop web-based accession of the genetic data for availability to the research community
- Investigate the population genetic structure of helminth parasites (and of their intermediate hosts/vectors where relevant) collected from different countries as well as from different geographical areas and transmission zones within countries, with particular emphasis on the development of tools to detect and monitor selection and spread of drug resistant genotypes

**2. Parasite Immunology**

- Conduct systematic investigations of the relative impact of single and multiple-species infection on innate and acquired immunity, immunopathology and morbidity, and the effects of single or integrated disease control programmes on these processes
- Assess the efficacy and seroconversion rates of current and anticipated vaccination programmes (including the deployment of novel anti-tuberculosis and anti-malarial vaccines) in populations with a range of helminth infection prevalence and intensity (from non-endemic to hyperendemic) levels, different helminth infections and diverse immune response profiles
- Investigate evolutionary trade-offs in host immune responses and helminth parasites’ manipulation of such responses. Use this knowledge for rational design of anti-helminth vaccines and prevention of unintended outcomes (increased transmission and/or morbidity)
- Increase R&D on a selection of the most promising vaccine candidates, including anti-schistosome and anti-filarial vaccine antigens, validating their efficacy in animal models and progressing their development for clinical trials and ultimately human use

**3. (Vertebrate) Host–Parasite Interactions and Pathogenesis**

- Study proteomics of host-parasite interface, including excretory-secretory antigens, surface antigens, host ligand-parasite receptors/host receptors-parasite ligands, and identify and characterized released factors that cause pathology to host cells including products that cause carcinogenesis. Develop high-throughput transcriptome analyses for the identification of host responses to helminth infection both *in vitro* and *in vivo* to further understanding of molecular pathogenesis
- Determine the effect of the helminth-activated innate immune responses on target cells, particularly in local responses and the adaptive immune response of the host, and assess the role that innate or adaptive responses to helminth parasites play in the pathogenesis of inflammation and immunopathology
- Explore the effects of repeated infections and treatments on innate and adaptive immune responses in the induction of pathology (including cancer)
- Identify pathogenic parasite strains using microsatellite or other DNA markers in certain diseases and different geographical areas, and test their pathogenicity *in vitro* and *in vivo* (the latter in suitable experimental animal models)
- Use genetic epidemiology to define potential heritable determinants of host susceptibility to helminth infection or severe disease, and use new molecular technologies for assessing and monitoring predisposition to, and occurrence or progression of helminthic diseases

**4. Invertebrate Host–Parasite Interactions and Transmission Biology**

- Investigate vector–parasite interactions (and their underlying mechanisms) in the most important combinations that participate in transmission in natural settings (including inter-specific interactions between helminth species and other parasites within the vectors/intermediate hosts)
- Conduct research on how the *Wolbachia* endosymbionts of arthropod vectors and the *Wolbachia* endosymbionts of the filarial nematodes influence the competence of the vectors and the transmission biology of the parasites
- Investigate trematode–snail–vertebrate intermediate host interactions in Asian liver flukes (e.g. study metacercarial / fish host interactions)
- Develop novel molecular-based methodologies to quantify infectivity of vectors, taking into account parasite genetic variation and geographical diversity, that will help evaluate MDA interventions and determine programme end-points
- Develop novel approaches for the detection of insecticide/molluscicide resistance
